# Integrating Inland and Coastal Water Quality Data for Actionable Knowledge

**DOI:** 10.3390/rs13152899

**Published:** 2021-07-23

**Authors:** Ghada Y.H. El Serafy, Blake A. Schaeffer, Merrie-Beth Neely, Anna Spinosa, Daniel Odermatt, Kathleen C. Weathers, Theo Baracchini, Damien Bouffard, Laurence Carvalho, Robyn N. Conmy, Liesbeth De Keukelaere, Peter D. Hunter, Cédric Jamet, Klaus D. Joehnk, John M. Johnston, Anders Knudby, Camille Minaudo, Nima Pahlevan, Ils Reusen, Kevin C. Rose, John Schalles, Maria Tzortziou

**Affiliations:** 1 Deltares, Boussinesqweg 1, 2629 HV Delft, The Netherlands; 2 Delft Institute of Applied Mathematics, Delft University of Technology, Mekelweg 5, 2628 CD Delft, The Netherlands; 3 U.S. Environmental Protection Agency, Office of Research and Development, Washington, DC 20460, USA; 4 Global Science & Technology, 7855 Walker Drive, Suite 200, Greenbelt, MD 20770, USA; 5 EAWAG, Swiss Federal Institute of Aquatic Science and Technology, 8600 Dübendorf, Switzerland; 6 Cary Institute of Ecosystem Studies, Millbrook, NY 12545, USA; 7 School of Architecture, Civil and Environmental Engineering, Ecole Polytechinque Fédérale de Lausanne, 1015 Lausanne, Switzerland; 8 EAWAG, Swiss Federal Institute of Aquatic Science and Technology, 6047 Kastanienbaum, Switzerland; 9 UK Centre for Ecology & Hydrology, Penicuik, EH26 0QB, UK; 10 VITO Remote Sensing, Boeretang 200, 2400 Mol, Belgium; 11 Earth and Planetary Observation Science (EPOS), Biological and Environmental Sciences, Faculty of Natural Sciences, University of Stirling, FK9 4LA Stirling, UK; 12 Univ. Littoral Cote d’Opale, Univ. Lille, CNRS, UMR 8187, LOG, Laboratoire d’Océanologie et de Géosciences, F 62930 Wimereux, France; 13 CSIRO Land and Water, Clunies Ross Street, Canberra ACT 2601, Australia; 14 Department of Geography, Environment and Geomatics, University of Ottawa, 60 University, Ottawa, ON K1N 6N5, Canada; 15 NASA Goddard Space Flight Center, 8800 Greenbelt Road, Greenbelt, MD 20771, USA; 16 Science Systems and Applications, Inc., 10210 Greenbelt Road, Lanham, MD 20706, USA; 17 Department of Biological Sciences, Rensselaer Polytechnic Institute, Troy, NY 12180, USA; 18 Creighton University, 2500 California Plaza, Omaha, NE 68178, USA; 19 The City College of New York, City University of New York, New York, NY 10003, USA

**Keywords:** water quality, remote sensing, lake, estuary, coastal, sensors, management, interoperability, integration

## Abstract

Water quality measures for inland and coastal waters are available as discrete samples from professional and volunteer water quality monitoring programs and higher-frequency, near-continuous data from automated in situ sensors. Water quality parameters also are estimated from model outputs and remote sensing. The integration of these data, via data assimilation, can result in a more holistic characterization of these highly dynamic ecosystems, and consequently improve water resource management. It is becoming common to see combinations of these data applied to answer relevant scientific questions. Yet, methods for scaling water quality data across regions and beyond, to provide actionable knowledge for stakeholders, have emerged only recently, particularly with the availability of satellite data now providing global coverage at high spatial resolution. In this paper, data sources and existing data integration frameworks are reviewed to give an overview of the present status and identify the gaps in existing frameworks. We propose an integration framework to provide information to user communities through the the Group on Earth Observations (GEO) AquaWatch Initiative. This aims to develop and build the global capacity and utility of water quality data, products, and information to support equitable and inclusive access for water resource management, policy and decision making.

## 1. Introduction

Considered the most valuable natural resource, water is essential to human life and the health of the environment. Accurate monitoring and forecasting of the quality of inland waters, defined here as lakes, reservoirs, estuaries, and coastal marine waters, is fundamental for developing sustainable water resource management strategies and ensuring the health of communities, ecosystems, and economies [1]. Yet, our knowledge of water quality is often disconnected in time and space across various measurement techniques and platforms that may fail to capture dynamic ecosystem changes. This disconnection represents an inefficiency and redundancy in research and monitoring activities. A major challenge for water resource management is how to integrate multiple sources of water quality data and indices into usable information of economic, social, infrastructural, environmental, or administrative value [2]. In addition, realizing the full application potential of emerging technologies requires solutions for merging various measurement techniques and platforms into useful information for actionable management decisions, requiring effective communication between data providers and water resource managers [3].

**Figure 1.**
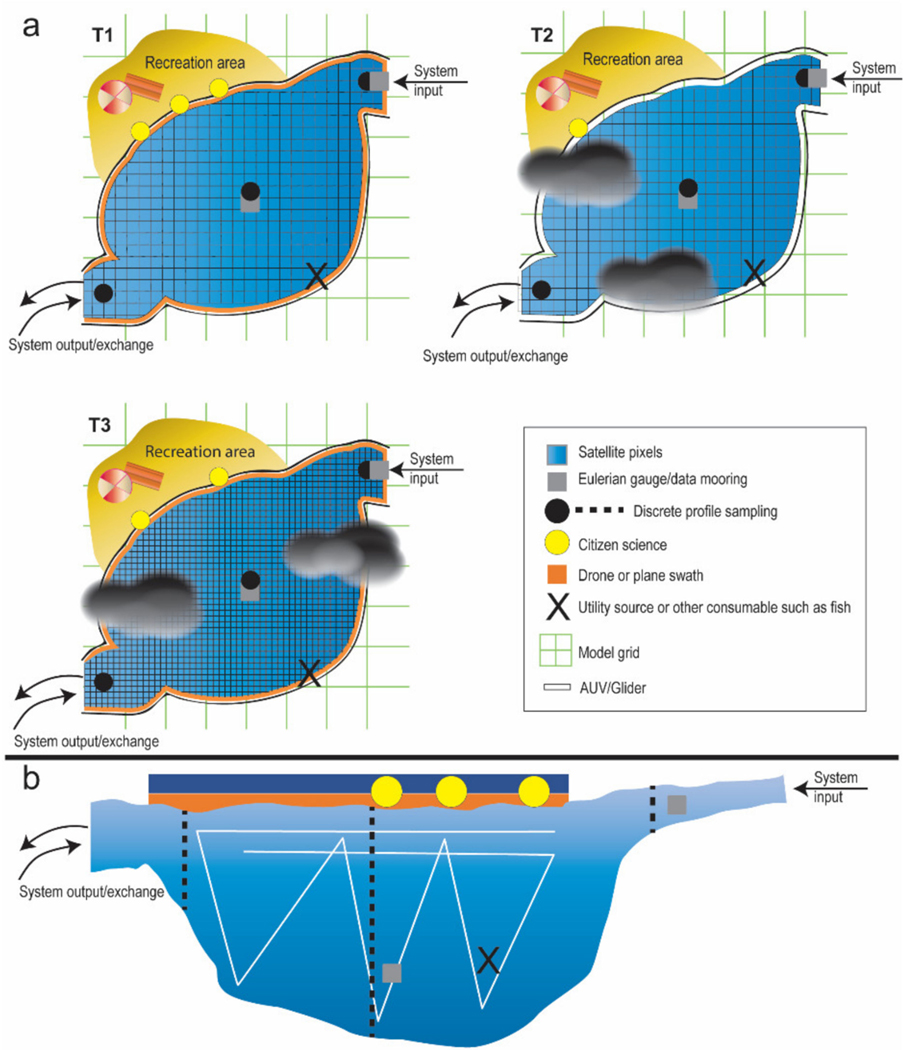
Conceptual diagram of an integrated sampling scheme in a generic waterbody that may represent a lake, reservoir, estuary, or coastal water. Complementary monitoring approaches provide information that can be used to address environmental, recreational, and consumable (food/drink) water quality and quantity. (**a**) surface view of sampling represents some of the spatial and temporal trade-offs between approaches, and (**b**) represents a vertical depth profile view.

The Group on Earth Observations (GEO) AquaWatch is a community of practice that aims to develop and build the global capacity and utility of water quality data, products, and information to support water resources management and decision making. GEO AquaWatch targets a wide range of stakeholders, from economists to scientists, to industry representatives, resource managers, nongovernmental groups, and policy makers at the local, regional and national levels (https://www.geoaquawatch.org/, accessed on July 2021). Integration of inland and coastal water quality data products are required for the successful implementation of the United Nations 2030 Sustainable Development Goals (SDG) and the Sendai Framework for Disaster Risk Reduction. Water quality monitoring is relevant toward SDG 6.3 to improve water quality by reducing pollution, eliminating dumping and minimizing release of hazardous chemicals and materials; SDG 6.6 to protect and restore water-related ecosystems; SDG 14.1 to prevent and significantly reduce marine pollution of all kinds; and for disaster risk reduction water quality monitoring and forecasting is critical for the development and assessment of safe water and sanitation systems. Here, we provide a conceptual approach developed through GEO AquaWatch for integrating data products to create water quality nowcasts (very short time forecast) and forecasts. Some of the main challenges in water quality data integration are discussed in Section 2. Integrated data products are derived from data assimilation of observations collected over various scales in space and time and from a range of platforms, discussed in Section 3. These data (Figure 1) include in situ discrete samples, from professional and citizen science/volunteer (or, community) monitoring programs, automatic in situ sensors, satellite, drone, or aircraft remote sensing, and model outputs. The actual integration process is addressed with a recommendation for a framework to take forward, discussed in Section 4.

**Figure 2.**
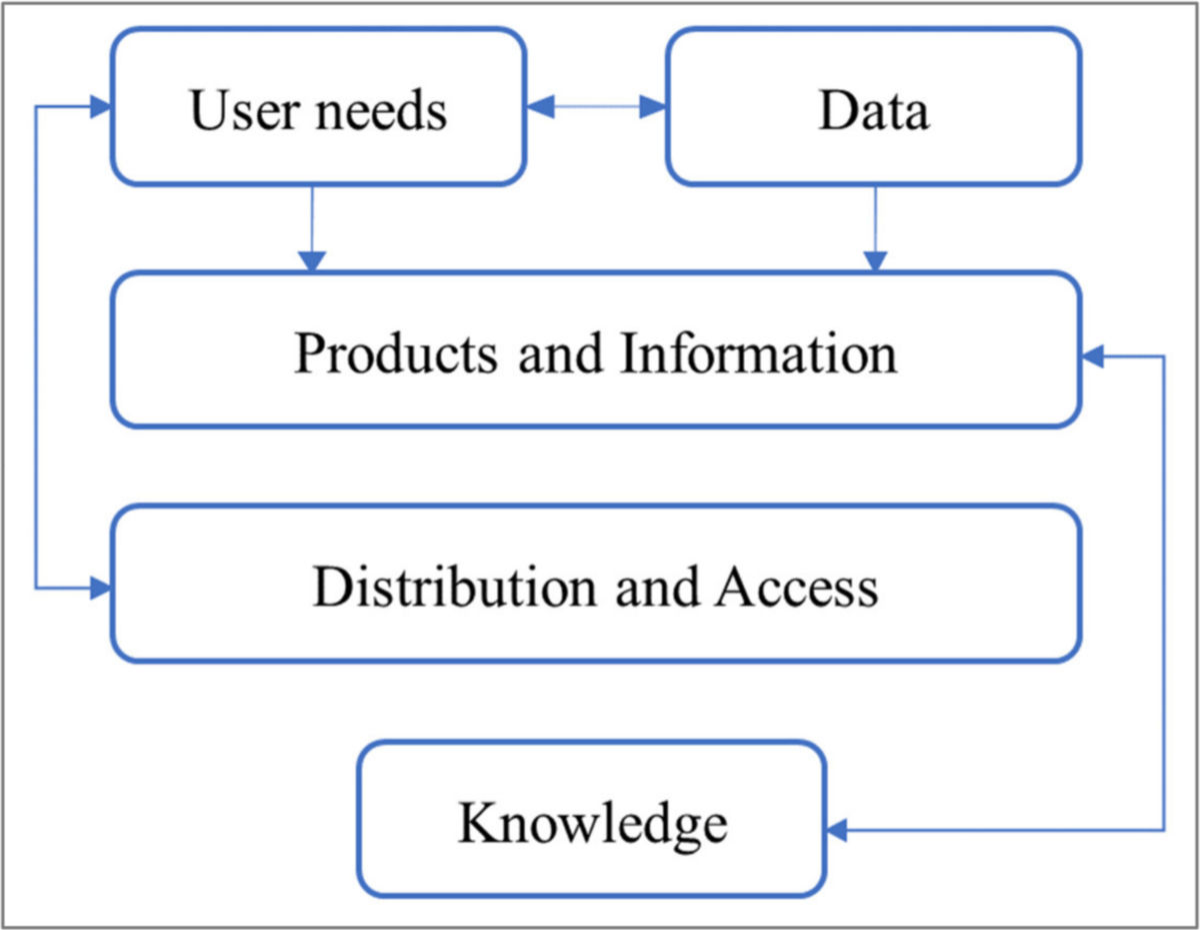
Schematic of the iterative steps GEO AquaWatch has developed to transform stakeholder needs into actionable knowledge.

Beyond water quality data integration, translating this information to actionable knowledge and linking to stakeholder needs requires an iterative approach. GEO AquaWatch has developed such an approach based on an iterative development model used in the climate science community [4–6]. Briefly, the iterative development model enables research agendas to be created and modified by both scientists and stakeholders [5]. GEO AquaWatch defined the iterative development model into five stages: (1) defining end-user needs; (2) generating or obtaining data required to meet the needs; (3) aggregating or transforming the data into usable products and information; (4) distributing and enabling access to the products and information; and, finally, (5) translating the products and information into actionable knowledge through data assimilation of water quality derived from multiple space and time observations and platforms (Figure 2).

## 2. Gaps and Challenges

Major challenges in data integration for water quality nowcasting and forecasting include data availability, interoperability, and reliability [7–9]. This is true for either fundamental trend analyses or more sophisticated model calibration and validation. Spatially and temporally sparse sampling, various methods, and protocols for any given parameter; missing ancillary data; incomplete or nonexistent metadata; and difficulty in accessing data portals all contribute to uncertainties [7] and lead to questions about the robustness of the data that do exist, such as: do we know the metadata provenance and trustworthiness? Do we know that proper quality controls were implemented, such as standard calibrations? Answers to these questions and others are critical for determining whether data are findable, accessible, interoperable, and repeatable [10], which are necessary to ensure, for example, that the science used in decision making is based on the best available and trusted data.

There is also a tendency for sampling programs to neglect exchanges and interactions across systems. For example, sampling only the receiving water body, such as an estuary or lake, but not the supplying rivers, streams, and groundwaters prevents consistent and comprehensive monitoring of water quality across the aquatic continuum and hinders the assessment and understanding of processes and pressures that operate across these interconnected systems. Evaluating such systems requires a better mechanistic understanding of the fluvial continuum from catchment to receiving water body. Evaluation must also include the preconditioned natural forces of change acting on systems to assess their resilience to absorb human-induced perturbations and extreme events for effective management, and to avoid tipping points [11]. This requires long term data streams that are consistent both spatially and temporally, which can be a challenge to find among government agencies. Data continuity and fidelity can also be hindered by short project funding cycles that have long term consequences.

Developing countries and remote locations often have minimal technical or institutional capacity to monitor water quality, manage the data, and report results [12]. This compounds the challenges of moving toward integrated water quality measures. Although international standards for water quality assessment (e.g., ISO—International Organization for Standardization) and data sharing, including metadata (e.g., FGDC—Federal Geographic Data Committee, ODC—Open Data Cube), are in place, no single standard is widely adopted and followed, thereby contributing to this challenge All this can lead to large observational gaps of robust water quality data, needed to underpin water management decisions. One solution is to rely on innovative monitoring approaches, such as via the integration of satellite and drone remote sensing, citizen science, and models. A demonstration project in Bangladesh recently highlighted the impact of the use of satellite data by decision makers, where coordinated or centralized capabilities ensured users were trained for the use of satellite information and understood the limitations of different satellite products [13]. Global provision of water quality data often requires the integration of different types of data from different sources for use by different communities. Scattered initiatives provide databases and platforms but lack concrete sustainable support to maintain and widely use those infrastructures. A global platform will enable the community to access information globally, on which analysis can be carried out, decisions can be made, and policy questions can be answered.

## 3. Overview of Observational Methods and Platforms

**Figure 3.**
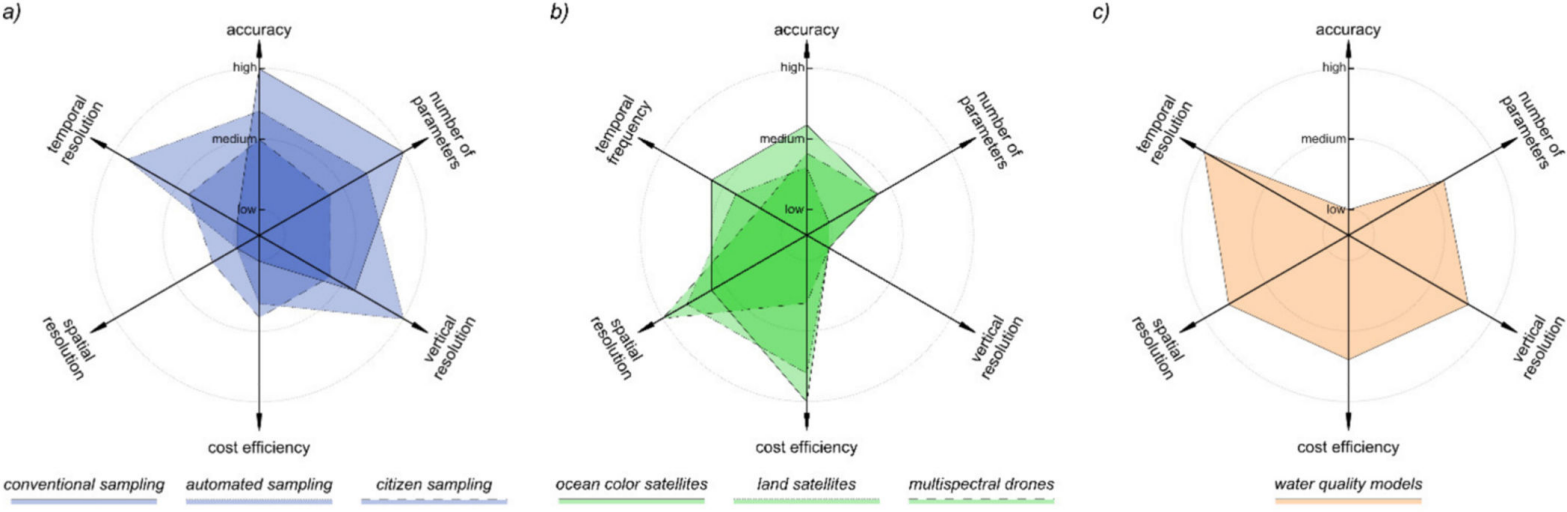
Schematic representation for the combination of (**a**) discrete, continuous, and citizen science measures combined with (**b**) remote sensing, and (**c**) modelling data. The combination provides complementary trade-offs of accuracy, number of parameters provided, spatial resolution, temporal frequency, and cost efficiency.

Measurements from different observing platforms, sensors, and data processing systems have different capabilities and characteristics, including differences in accuracy, number and type of parameters provided, temporal frequency, spatial coverage and resolution, and cost efficiency (Figure 3). The physical, chemical, or biogeochemical parameters of interest are manifold, but preference is increasingly given to those advanced as Essential Biodiversity, Climate or Water Variables [14–16]. In situ measurements often have high vertical (water column) resolution but low horizontal resolution, here defined as the capability to distinguish horizontal gradients, whereas, ocean color satellite data provide the converse. For example, in monitoring a river outflow or wastewater discharge, field sampling and sensors can provide data on many parameters at high frequency, potentially at high spatial resolution (in centimeters) and in both horizontal and vertical dimension, but low spatial coverage, whereas a satellite sensor (10 to 30 m) or drone (10 to 100 cm) may provide relevant information at sufficient spatial resolution and high spatial coverage, but miss variability in the vertical dimension. Another example may be a lake with a public drinking water intake. Here, an ocean color satellite may be useful for monitoring the entire lake, in situ sensor may benefit for high frequency sampling at the depth of the intake, discrete sampling enables event-driven responses, and models may be useful to understanding how upstream anthropogenic stressors may impact this resource and test scenarios to protect and restore water quality. If these sources are properly integrated, as proposed, many of the trade-offs and strengths are mitigated, enabling a more comprehensive toolbox for monitoring and interpreting water quality.

### 3.1. Discrete Sampling

Discrete sampling has been used for decades to obtain information about physical, chemical, and biogeochemical parameters related to water quality [17]. Once on site, it is easy to deploy instruments, such as Secchi discs, optical sensors, conductivity, temperature, and depth sondes, and to collect discrete water samples using collection bottles, such as Niskins. These measurements (surface and vertical profiles) are made from the shoreline, boats, buoys, and automated vehicles at a single location or across transects, depending on the extent of the water body of interest and the aim of the sampling. The measurement protocols generally are very well defined, and the instruments are easy to handle and cost-effective [1,18–26]. After collection, water samples either are processed or filtered separately and typically preserved for further analysis in laboratories. The measured parameters provide information about autochthonous processes, the consequences of external inputs to these water bodies (salinity, pH, nutrients, particulate organic and suspended matter), and on the functioning of the ecosystem (dissolved oxygen and plankton biomass), leading to a comprehensive and extensive suite of analytes to assess the health of the water body. Grassroots networks and regional, and national programs have been developed for measuring and monitoring essential variables in nearshore coastal, estuarine, and inland waters (for example, the French monitoring network Service d’Observation en Milieu LIT-toral (SOMLIT); the Global Lake Ecological Observatory Network (GLEON; [27]); and the European Ocean Observing System (EOOS)).

However, discrete sampling can be costly in terms of human resources and use of equipment. Measurements are typically local and sampled over a very limited area and a short period. These measurements typically are limited to bi-weekly, monthly, or even quarterly surveys, often missing diurnal or day-to-day variability. This low frequency means that measurements rarely characterize the variability of the water body of interest adequately and may be insufficient to detect spatial variability within the waterbody and trends over time unless conducted for many years. The water body also may be difficult to access, for example when far from accessible infrastructure, limiting the number of surveys and increasing the survey costs. There is often a lack of cyber-infrastructure to enable near real-time sharing of discrete data, whereas different instruments, sampling protocols, and quality assurance procedures are used across laboratories, organizations, and countries. There are also limited coordinating monitoring networks globally, such as the European Global Ocean Observing System (EuroGOOS) or the Fiducial Reference Measurements for Satellite Ocean Colour (FRM4SOC) [28] across Europe, the National Water Quality Monitoring Network, or the Global Environment Monitoring System for Freshwater (GEMS/Water) or the National Water Quality Program in the United States.

### 3.2. Continous Sampling

**Figure 4.**
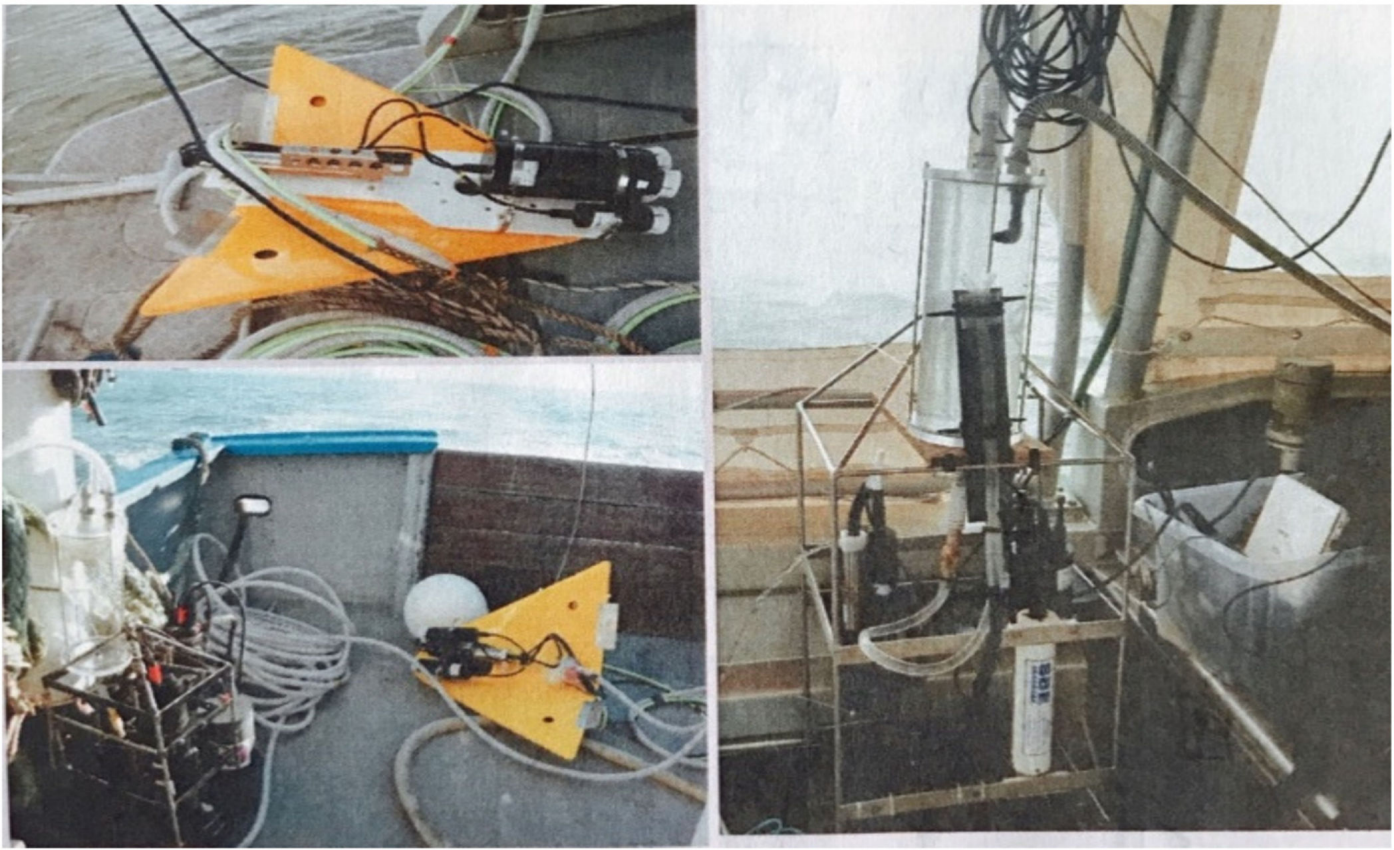
Bot-wing equipped with a CTD and optical sensor for high spatial lateral and depth measurements (in-flow).

**Figure 5.**
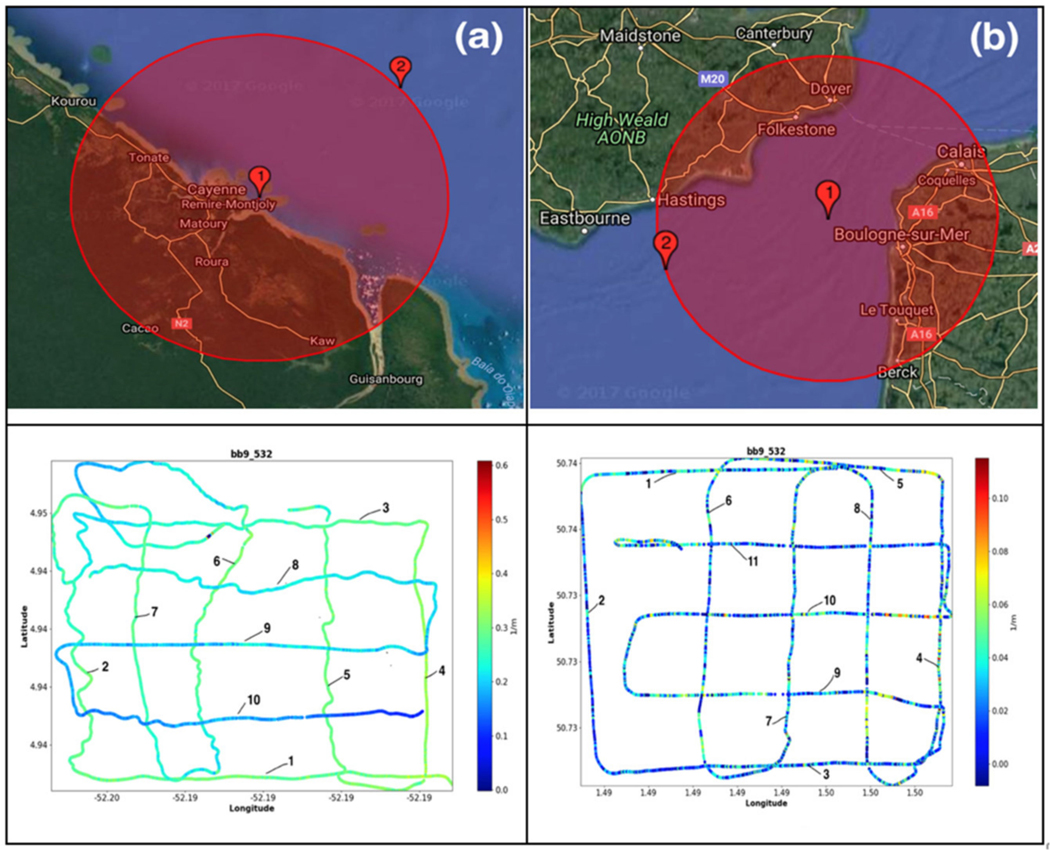
Examples of in-flow measurements of the back-scattering coefficients in French Guyana (**a**) and the Eastern English Channel (**b**).

Continuous sampling helps overcome the limitations of discrete sampling by providing high frequency measurements over time [29,30]. Such systems have been used for decades to measure parameters, such as salinity, temperature, depth (CTD), and chlorophyll fluorescence (ARGO floats; [31]). Recent improvements in technology have enabled the integration of optical sensors for measurements of irradiance, spectral absorption, fluorescence, and backscattering from buoys [32] from flow-through systems installed on-board ships (Figure 4) [19], and from profiling floats (BCG-Argo; [33]), or gliders [34]. These instruments provide high spatial resolution measurements, in both the lateral and vertical dimensions, with scales between 10 and 100 m depending on the speed of the ship and integration-time of the instruments (Figure 5) [35].

**Figure 6.**
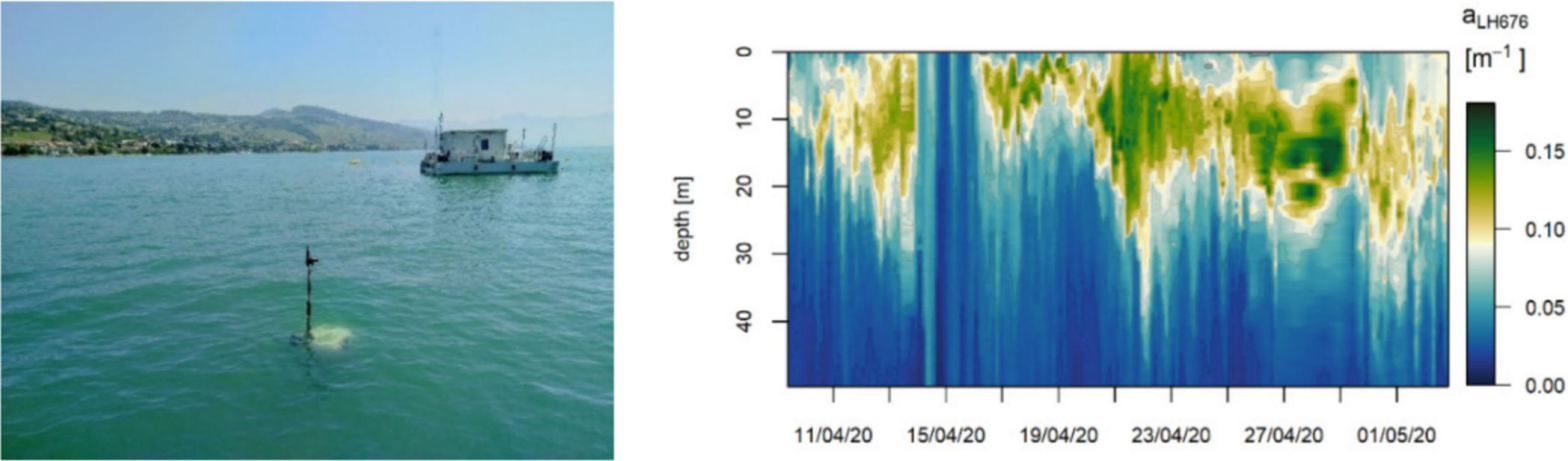
High resolution profiles of absorption line height peak at 676 nm measured between April 9 and May 2, 2020 with a WetLabs ACS mounted on a Thetis autonomous profiler near the platform LéXPLORE in Lake Geneva (Switzerland-France). Every 3 h, the Thetis profiles the top 50 m of the water column and produces various bio-optical and physical data at a centimeter resolution.

Fixed platforms provide a different type of continuous sampling through a wide variety of techniques typically used with buoys or on hard-mounted platforms. The AERONET-OC network provides continuous and automatic measurements of the remote sensing reflectance in coastal waters [36]. GLEON (www.gleon.org, accessed on July 2021) has evolved to encompass nearly 100 of similarly purposed buoys across the globe. In addition to the data provided, continuous sampling also benefits the validation of remote sensing products. The dynamic changes that can occur in water quality variables throughout the day are shown in Figure 6. Another example of a single-lake system is Lac Léman Exploration (LéXPLORE; https://lexplore.info/, accessed on July 2021), which is an experimental platform deployed in 2019 on Lake Geneva and equipped with instruments that measure coupled biogeochemical and physical processes at fine temporal and spatial resolution. The platform aims to provide a wide range of open access in situ data (from bio-optical to physico-chemical measurements and samples) to foster interdisciplinary research, inform stakeholders on the lake status, and communicate science to the public. The temporal resolution and the variety of data measured at such platforms make them effective for validating remote sensing measurements and model simulations.

### 3.3. Remote Sensing

#### 3.3.1. Passive Remote Sensing

Space-based, optical observations provide a unique capability to obtain spatially resolved water quality information over large geographical scales and at relatively high temporal frequencies [37–40]. The use of satellite data for mapping water quality parameters in inland and coastal systems can be traced back to the early 1970s, when the Landsat satellite program captured the first observation of ocean color from space. Over recent years, the exploitation of satellite data has increased significantly, as the spatial, spectral, and radiometric characteristics of satellites with ocean-color capability have improved considerably.

Space agencies and, increasingly, private companies operate several satellite missions for aquatic and terrestrial environmental monitoring. Remote sensing systems provide calibrated top-of-atmosphere radiance or reflectance with varying spectral, temporal, and spatial sampling resolutions. This radiometric signal enables a direct retrieval of optically active water quality properties, such as phytoplankton, nonalgal particles (or turbidity), and colored dissolved organic matter, as well as physical properties, such as surface temperature [18]. Polar-orbiting, multispectral ocean color satellite missions provide high temporal revisit data (typically global coverage at 1 to 3 days) at moderate spatial resolutions (0.3 to 1 km), and, as such, these sensors are well suited to the operational monitoring of large lakes and coastal waters. Ocean color satellite band settings are optimized specifically for aquatic applications, and, although challenges remain, significant research efforts have been invested in the development of improved algorithms for the atmospheric correction and retrieval of water quality information from such data [41–49]. The design of new ocean color sensors with extended spectral range (UV to SWIR) and hyper-spectral capabilities, such as PACE (Plankton, Aerosol, Cloud, ocean Ecosystem), and improved temporal resolution (multiple observations per day), such as the geosynchronous GLIMR (Geosynchronous Littoral Imaging and Monitoring Radiometer), will enable the identification and tracking of advanced aquatic biological and ecological indicators to guide sustainable management in inland and coastal waters [50,51]. For application to inland and coastal waters, measurements from ocean color sensors are often complemented by satellite optical imagery from high spatial resolution sensors primarily designed for terrestrial observations (such as the Landsat-8/OLI—Operational Land Imager (OLI) and the Sentinel-2 MSI—MultiSpectral Instrument). Although the design of these sensors is not optimal for the observation of inland and coastal waters, with coarser spectral, radiometric, and temporal resolutions compared with heritage and existing ocean color missions (e.g., VIIRS—Visible Infrared Imaging Radiometer Suite), their higher spatial resolution (10 to 30 m), enables to observe small-scale variability in optically active water quality parameters, such as chlorophyll-*a* and total suspended matter in waters. The recent emergence of microand nano-satellite constellations (e.g., PlanetLabs Doves; [52]) that provide data at even higher spatial (<5 m) and temporal resolutions (daily) hold considerable promise for water quality monitoring, particularly for smaller waterbodies, although considerable research is required to evaluate their radiometric stability [53] and develop robust approaches to atmospheric correction and retrieval of water quality parameters. The CubeSat mission SWEET (Sweet Water Earth Education Technologies) [54] and SeaHawk (https://uncw.edu/socon/seahawk.html, accessed on July 2021), addressing the gaps of lack of dedicated missions and high costs for vessels, is also a promising new technology for the monitoring of water quality with a revisit time of 3 to 5 days. Complementing these systems are a number of geostationary ocean color sensors (e.g. GOCI—Geostationary Ocean Color Imager) and meteorological sensors that demonstrated potentiality to observe turbidity dynamics (e.g. SEVIRI—Spinning Enhanced Visible and InfraRed Imager), and have the advantage of obtainaing hourly to daily data allowing the investigation of ocean properties variations over a day [55–58].

Integrating data from different sensors and missions is imperative to resolve the physical and biological complexity of most inland and coastal waters because of the combined requirements for high spatial, temporal, and spectral resolution data [59]. The observational characteristics of satellite data are highly complementary to those of in situ monitoring programs. They also enable indicative monitoring for a larger number of lakes where no other data sources are available. For example, satellite sensors are often the only means of acquiring regular water quality information in regions where issues of accessibility and lack of resources limit in situ monitoring, and make these regions scarcely sampled compared with more economically developed and more densely populated regions. Providing this synoptic perspective is critical to resource planning, decision making, and improved model predictions of future change [60,61].

Yet, satellite observations alone cannot provide all information needed by policy makers and environmental managers to address inland and coastal water quality issues [62–65]. The limitations of satellite remote sensing for water quality monitoring are the range of derived parameters and the inability to retrieve information from depth [66]. The performance of atmospheric correction models and algorithms in the optically complex waters also remains an issue for some applications where highly accurate products are required. Other much-quoted technical limitations concern spatial resolution and sampling gaps due to cloud coverage [67]. Slow adoption of satellite remote sensing data for water quality monitoring is also because of practical reasons, such as data continuity, and retrieval accuracy requirements [3]. Validation of satellite data products for inland and coastal waters is a challenge for scientists and concern for stakeholders. There are two aspects to this challenge for scientists: (1) acquiring and/or accessing suitable in situ data for calibration and validation of satellite measures and (2) minimize the propagation of errors from in situ data to satellite measures.

#### 3.3.2. Active Remote Sensing

Some of the weaknesses of optical images can be compensated by active remote sensing observation. Synthetic Aperture Radar (SAR) has been used successfully to quantify physical properties of seawater such as biogenic films, phytoplankton scums, and to detect oil spills and bathymetric features in shallow waters [68,69]. SAR provides high spatial resolution (10 to 20 m) measurements of the backscatter signal of the ocean surface with a low frequency (typically 12 to 35 days). The use of SAR can be traced back to 1992, with the launch of ERS-1. Since then, several technological advances, both in spatial and temporal resolution, have been achieved making the use of radar remote sensing an additional tool to complement optical remote sensing for the monitoring of the health of a water body. Active sensors measuring through the microwave portion of the electromagnetic spectrum enable the acquisition of images day and night regardless of weather conditions. Lately, LiDAR (Light Detection and Ranging) method has attracted a lot of attention [70–73], as it can overcome some limitations of optical imagers only day-time observations of near-surface features under clear sky conditions. Airborne LiDAR has been used extensively in coastal waters for studying fish schools [74], and phytoplankton layers [75], and geomorphic changes in shorelines and sedimentary environments. Studies using spaceborne LiDAR (CALIOP) have shown that it is possible to estimate the backscattering coefficient at 532 nm over the global ocean [76–78]. Using the ICESat-2 spaceborne LiDAR, Lu et al. [79] retrieved two-dimensional (depth-resolved) distributions of particulate backscattering (at 532 nm), linking to particulate organic carbon (POC) and phytoplankton carbon biomass distributions in the coastal Antarctic ocean. Originally designed for monitoring the ocean and successively for mapping ice sheets and sea ice, the recent advances in radar altimetry technology have enhanced the resolutions and accuracy of altimetric data making them an interesting alternative for inland water measurements [80]. Radar satellite altimetry provides information on the Earth’s surface dynamical features of heigh sea and its changes over time. Crucial for the regional and global monitoring of sea level rise impact, radar altimeter measurements provides useful information for mapping a wide range of parameters including sea surface height, sea surface wind speed and significant wave height, but also vertical land motion estimating, bathymetric computations, and marine geoid modeling [81,82]. Altimetry data have also demonstrate to improve the estimation of lake ecological status and river discharge when merged with optical satellite imagery [83,84] although measurements for small-medium rivers remains challenging due to the poor spatial sampling and temporal resolution of satellite altimetry [85].

Such information from new active sensors, including airborne and space-based LiDAR, would help improve understanding of water quality and ecological changes in inland and coastal waters and would complement measurements from high spectral, spatial, and temporal imaging technologies.

#### 3.3.3. Drone and Aircraft Systems

The technology for smaller, portable, remotely piloted aircraft systems (RPAS), also known as unmanned aircraft systems (UAS) or, more commonly, drones, has evolved rapidly over the past two decades. These systems consist of both fixed-wing and copterstyle aircraft designs, aerial cameras and sensors, navigation systems that include electronics and GPS devices in the aircraft, combined with autopilot devices and/or controllers that communicate with the aircraft using radio frequencies. Current systems utilize cell phones and larger tablet devices with applications enabling a remote pilot to fly using the controller, supported by near real-time images returned from the aircraft’s camera. The aircraft can be flown manually or with a flight plan created by the drone manufacturer’s software (for example, DJI FlightPlanner), or with specialized applications (for example, Pix4D and Drone Deploy). Typically, hundreds of individual images, captured along gridded flight lines, are mosaicked as a 2-D GeoTIF and brought into raster-based remote sensing software for further analysis and generation of products.

Drone capabilities are especially helpful in nearshore and shoreline zones where satellite data suffer from mixed pixel and adjacency effects [86]. Drones can provide high spatial and, potentially, high temporal resolution information and are complementary to satellite and conventional manned airborne imagery platforms. The advantages of drones also include temporal and spatial flexibility, such as reaching inaccessible or dangerous locations [87]. Drones and aircraft can be deployed to fill monitoring gaps (e.g., on cloudy days), albeit at lower spatial extent, and to capture the spatial and temporal variations of water quality parameters within one flight or between flights, which is vital for comprehensive assessments of water bodies [62]. A well-integrated set of recorded tutorials and examples of drone applications for coastal research and monitoring are available from an October 2020 U.S.NOAA-sponsored virtual workshop (https://secoora.org/drones-in-the-coastal-zone-workshop/, accessed on July 2021).

A variety of conventional cameras and narrow-band imaging sensors (for example, Micasense Altum with RBG, Red Edge, NIR, and Thermal IR bands; Headwall Hyperspec sensors) have been adapted to drones and aircraft, enabling measurements across the solar reflective spectrum, as well as emissive thermal infrared. The following studies have used drones for a variety of water quality measurements: primary production and trophic state [88], harmful algal blooms [89,90], macrophytes [91,92], chlorophyll-a [93], total suspended matter (TSM) [94], and turbidity [95]. It is safe to assume that emerging technologies will provide more capabilities in monitoring water quality parameters. Several new drones are designed to be waterproof, enabling water landings and acquisition of shallow submersion underwater photography, or can be modified to collect water samples [96]. As an example, the Saildrone, an unmanned surface vehicle (USV; Saildrone, Inc.) [97,98] is capable of surfing on the water surface with sensors measuring atmospheric and aquatic parameters. For seawater, ocean currents, chlorophyll-a, colored dissolved organic matter (CDOM), backscatter coefficient, dissolved oxygen, sea surface temperature (SST), and sea surface salinity (SSS) can be measured. The system can be adapted to include other sensors. It is powered by wind and sun and can travel up to an average speed of around 1.25 ms^−1^. The Saildrone can perform autonomous long-range data collection (up to a 12-month mission, depending on biofouling). There are numerous commercially-available ASV and small autonomous underwater vehicles (AUV) for water water quality monitoring that are becoming available to all stakeholders. They have the potential to play a significant role in coastal and inland water quality monitoring in years to come. However, listing them here is beyond the scope of this paper.

Disadvantages of drones include frequent changes to codes, restrictions, and controlling legislation at the state and national levels [99]. Local and national laws now commonly require specialized training, certifications, and aircraft registration. Drone pilots must be aware of restricted airspaces. Obtaining permissions can sometimes be lengthy and complicated, although a new USA FAA flight approval process (LAANC) can provide dependable and rapid permission for flights in restricted airspaces using computer interactions between a certified operator’s flight planning software (for example B4UFLY) and local control tower operations. Operators should have good training and experience with dynamic weather conditions, natural and human-built obstacles, encounters and avoidance with other aircraft, birds, and protected wildlife species.

### 3.4. Volunteer Monitoring

Volunteer monitoring often referred to as citizen science, is the collection of scientific samples and data by members of the general public [100]. It is often carried out in collaboration with professional scientists to record biodiversity or monitor the environment. It can provide scientifically robust data at large spatial coverage and high frequency (e.g., CoCoRAHs, https://www.cocorahs.org/ accessed on July 2021 Weather Observation Website [WOW] https://wow.metoffice.gov.uk/, accessed on July 2021). Volunteer monitoring is also an effective approach for engaging the public in science and environmental monitoring, empowering individuals and communities to understand and manage the quality of their local environment [101]. Volunteer monitoring data can be complementary to remote sensing data, as they can be used together to provide independent cross-validation [34], although data quality of specific citizen science applications are not quite on par with scientific spectrometers [102].

The number of citizen science projects relevant to water quality monitoring is growing globally (e.g., see https://scistarter.org/, accessed on July 2021), and the development of mobile phone apps to gather geo-located data (e.g., Lake Observer, www.lakeobserver.org, accessed on July 2021) electronically makes integration with satellite data relatively easy. Some relevant examples of citizen science projects focused on water quality are listed in Table 1. Key challenges still exist in relation to quantifying the uncertainty of citizen science data, incorporating both measurement and sampling bias (locations and time/date of sampling), and also in recruitment and retention of citizen scientists [103,104], but integration with remote sensing and other in situ measurements could help overcome these challenges.

### 3.5. Models

A major benefit to the integration of modeling with monitoring datasets is the greater explanatory power over single observations alone [107]. Although remote sensing and most citizen science programs provide surface measures, and discrete and continuous sampling provide measures at depth, they all are limited to observation times. One- and three-dimensional integrated models provide time-stepped processes and distribution of water quality parameters laterally, as well in depth. Models provide tools quantifying rate processes and managing the ecosystem response to anthropogenic and natural conditions. Deterministic water quality models rely on a first correct description of hydrodynamic processes and conditions (e.g., stratification, shear, etc.) [108].

Compared with three-dimensional process-based models, one-dimensional (1-D) models have the advantage of being computationally inexpensive and can provide good estimates of the seasonal dynamics [109]. They are, thereby, excellent candidates for global scale and long-term climate change studies [110,111]. Yet, short-time responses and lateral variability remain out of the scope of such an approach. In three-dimensional (3-D) modeling, the horizontal and vertical variability are resolved over time. Extensive reviews of common ecosystem models are available elsewhere [112–114].

Ecosystem models often focus on plankton communities, food-web structures, and population dynamics. The combination of such coupled hydrodynamic-biological models with in situ observations and associated data analysis has been widely demonstrated [115–117]. A range of models are available for lake ecosystems [113,118], though, applying 3D hydrodynamic models in lake environments is more challenging than for coastal systems and the open ocean. The thinner and stronger stratification and weaker level of turbulence observed in lakes remain challenging to correctly reproduce with standard turbulence closure schemes [119]. The delay in adapting state-of-the-art techniques in lakes also is explained by the small size of research groups, however increasing, in limnology [120], focusing on particular lakes. Hydrodynamic and coupled biogeochemical outputs of the large-scale dynamics are improved by the addition of data assimilation from in situ and remotely sensed data [121–126]. However, fine-scale hydrodynamic processes and, even more so, biogeochemical processes become more diverse and difficult to resolve. Boundary effects, shallowness of the systems, and, thus, strong influence from the water-sediment interface, as well as submerged plant communities, macrophytes, and more variation in local meteorology, result in a far more dynamic environment.

There is also reduced availability of data to characterize lake or coastal ecosystems using only a few sampling points, often only a single site per lake, which might not be representative for the entire system [127,128]. Models that can integrate discrete, continuous, remote sensing, and citizen science data more readily may achieve desired forecasting systems for short- and long-term management of ecological, hydrological, and climatological impacts. The current challenge is the timely delivery of model result information to stakeholders for decision making. This is mainly achieved through online operational information portals [110,121]. Online operational portals aim at providing real-time monitoring and short-term information, which can be used on a variety of scales and problems for a variety of stakeholder interests.

## 4. Data Integration

GEO AquaWatch, long-recognized as the Global Water Quality Initiative with an extensive global network, coordinates an integrated approach to community stakeholder involvement, and its Knowledge Hub is recognized as a trusted global water quality data and information center. Ongoing efforts by GEO AquaWatch working groups and short-term, cross-cutting focus group activities have yeilded metadata compilations, best practices for data providers, aquatic analysis ready data recommendations, and expanded community-wide information-sharing. Transformational water quality knowledge is delivered to GEO AquaWatch stakeholders via our website and social media accounts, a successful and sustaining webinar series, topical workshops, and Initiative meetings. Printed and electronic work products, such as whitepapers, publications, and data products, services, and tools compiled by our diverse subject matter experts, are easy-access, community-wide resources serving the gamut of water quality users. Leveraging GEO AquaWatch’s association with other national and international water quality organizations amplifies best-practice messaging and enables broader user input.

Stakeholder engagement in the delivery of fit-for-purpose, open source products, meeting user needs, is foundational to GEO AquaWatch’s portfolio of projects. A highlight is it’s namesake Google Earth Engine project for inland and coastal waters whichcompiles a reference database of biogeochemical, inherent, and apparent optical properties that is essential for calibration and validation of satellite products. AquaWatch features a self-guided user interface with a nimble API that delivers chunk-sized data streams to the Google Earth Engine Servers.

To effectively achieve integrated inland coastal water quality management, users ideally inform their decision-making through combination of evaluating acute conditions and general trends in satellite-derived and in situ data, and utilizing iterative models; but we recognize many users lack one or more of these “three pillars” of data assimiliation and must otherwise rely upon the best available information. The complexity of environmental systems, and the need for meaningful information to be provided to stakeholders, requires the integration of data collected from different and heterogeneous sources. GEO AquaWatch’s Real Earth Portal, expected to debut in 2022, synthesizes multi-platform water quality data from disparate sources and over broad temporal-spatial scales accessible through the GEO AquaWatch website. Many hydrodynamic and ecological models have evolved as relevant predictive tools, and it is essential to accurately calibrate and validate them with available observations to validly represent the reality. Data fusion (DF) techniques have been used to maximally extract consistent spatial and temporal description of variables within hydrodynamic-biological models, cohesively and systematically, to produce more robust and informative datasets. DF algorithms include the wavelength-based method [129], the Jointly Sparse Fusion of Images (J-SparseFI) [130] designed for pan-sharpening fusion, and other algorithms [131,132]. The effectiveness of DF has been demonstrated in improving spatio-temporal distributions of total organic carbon (TOC) [133], chlorophyll-a concentrations, and water transparency [134], and the prediction of harmful algal blooms [135].

NASA-promoted STAR-FM software (https://www.ars.usda.gov/research/software/download/?softwareid=432, accessed on July 2021), designed for the fusion of Landsat and MODIS images, is available for direct implementation of fusion techniques at the pixel level [136]. Spatial and spectral information of the pixel is correlated and combined to better characterize the single pixel.

To refine model parameters and better align prediction with measurements, Data Assimilation (DA) techniques have been used [137]. DA enables automated calibration of models, as well as enhancing forecast capabilities of biogeochemical and hydrological models and improving ecosystem state assessments [138]. Among the most common DA methods, the Ensemble Steady State Kalman Filter (EnSSKF) [139] and the Ensemble Kalman Filter (EnKF) [140] have been widely and successfully used to improve the prediction of lake temperature [122], suspended particulate matter concentrations [141], water levels and currents [142], and algae and algal bloom dynamics [143,144] through the integration of remotely sensed and in situ data. Data integration techniques are variable and site specific depending at the phenomena at hand scale, this is beyond the scope of this paper and thus not addressed.

To promote and facilitate the use of DA methodologies, pre-fabricated algorithms and toolkits have been developed and made available from several working groups. These toolkits include the Parallel Data Assimilation Framework (http://pdaf.awi.de/trac/wiki, accessed on July 2021), the Data Assimilation Research Testbed (DART, https://www.image.ucar.edu/DAReS/DART/, accessed on July 2021), and the OpenDA (https://www.openda.org/, accessed on July 2021), which was used successfully for the automatic calibration of a water level forecasting model [145] and current and salinity profiles [146] and for the improvement of forecast accuracy of a water quality model [147,148].

With such a reservoir of ready-to-apply methodologies, the effort in using these techniques and applying them to current modeling is reduced. Nevertheless, the accurate calibration of system models requires high resolution description of the model inputs. These are not always readily available for many environmental systems because of the limitations of current measurement technologies. This remains a major challenge for DA applications in water quality management that can be overcome utilizing DF techniques to integrate, fuse, and harmonize in situ, remote sensing, volunteer monitoring, and modeled data.

### Integration Framework

**Figure 7.**
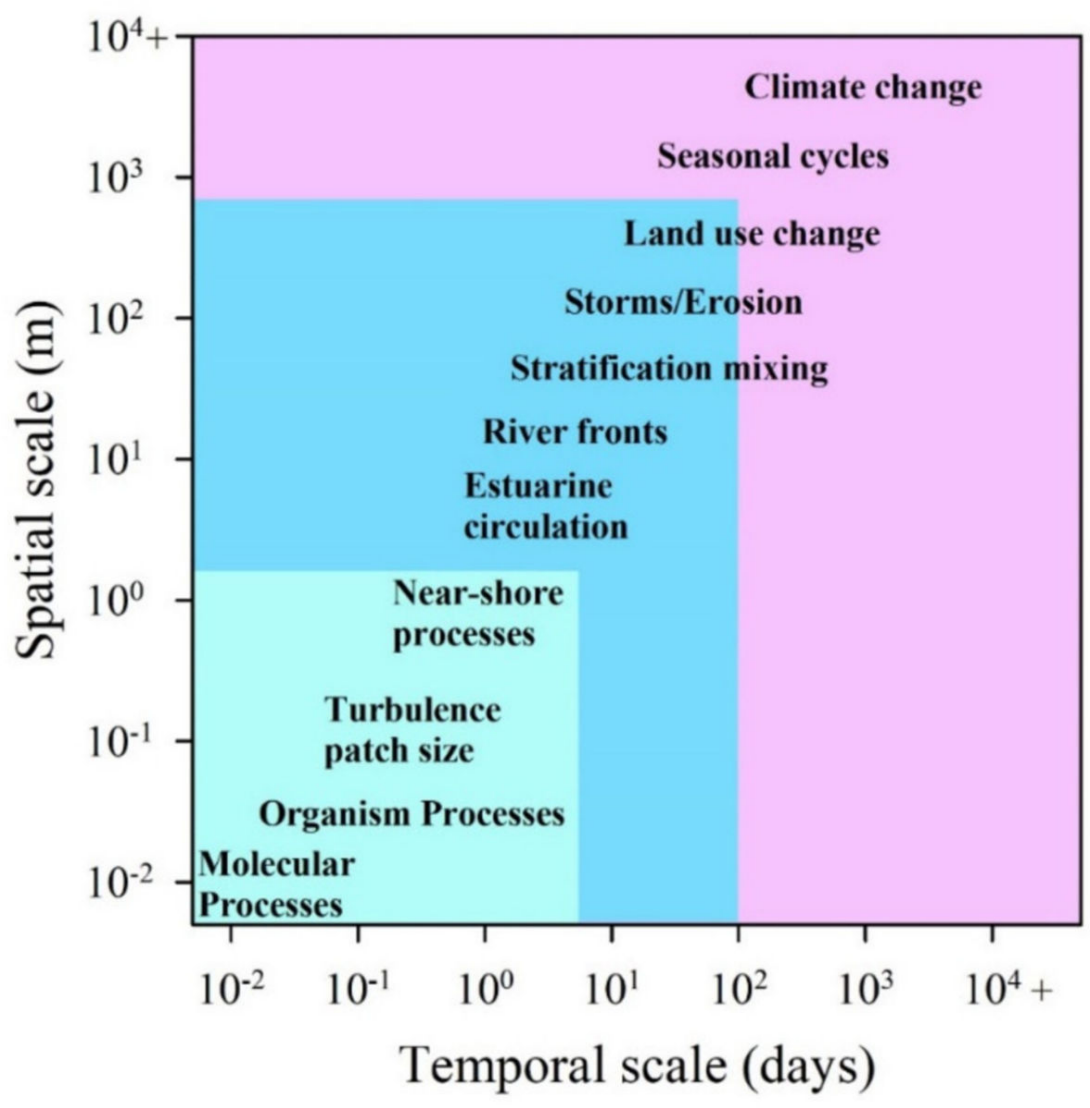
Continuum of temporal and spatial scale (resolution) and extent from molecular processes to large-scale hydrology and global climate processes.

The development of continuous, low-cost sensor networks, combined with wireless and cellular technology and big data analytics, has provided the opportunity to revolutionize decision making, using real-time data across wide ranges of spatial and temporal scales (Figure 7). Examples of real-time decision making from integrated data already include smart cities that manage municipal assets and optimize financial resource allocation through widespread use of data integration [149–151] and basin-wide water quality sensor integration, such as Clemson University’s Intelligent River Project in the South eastern United States [152]. The goal of smart cities is to utilize technology to manage infrastructure and resources efficiently. Sensor and citizen-provided data are analyzed to manage key infrastructure (e.g., buildings, road networks) and services (e.g., public transportation systems, waste management, law enforcement) for their communities. The smart city concept integrates information and communication technology to optimize the efficiency of city operations and enable real-time citizen feedback. The goal of Intelligent River is to deploy observation instruments that cover an entire river basin, such as the Savannah River and its reservoirs, with sensor technology, and combined with satellite data and additional continuous water quality monitoring sondes, in one easy-to-use data portal.

**Figure 8.**
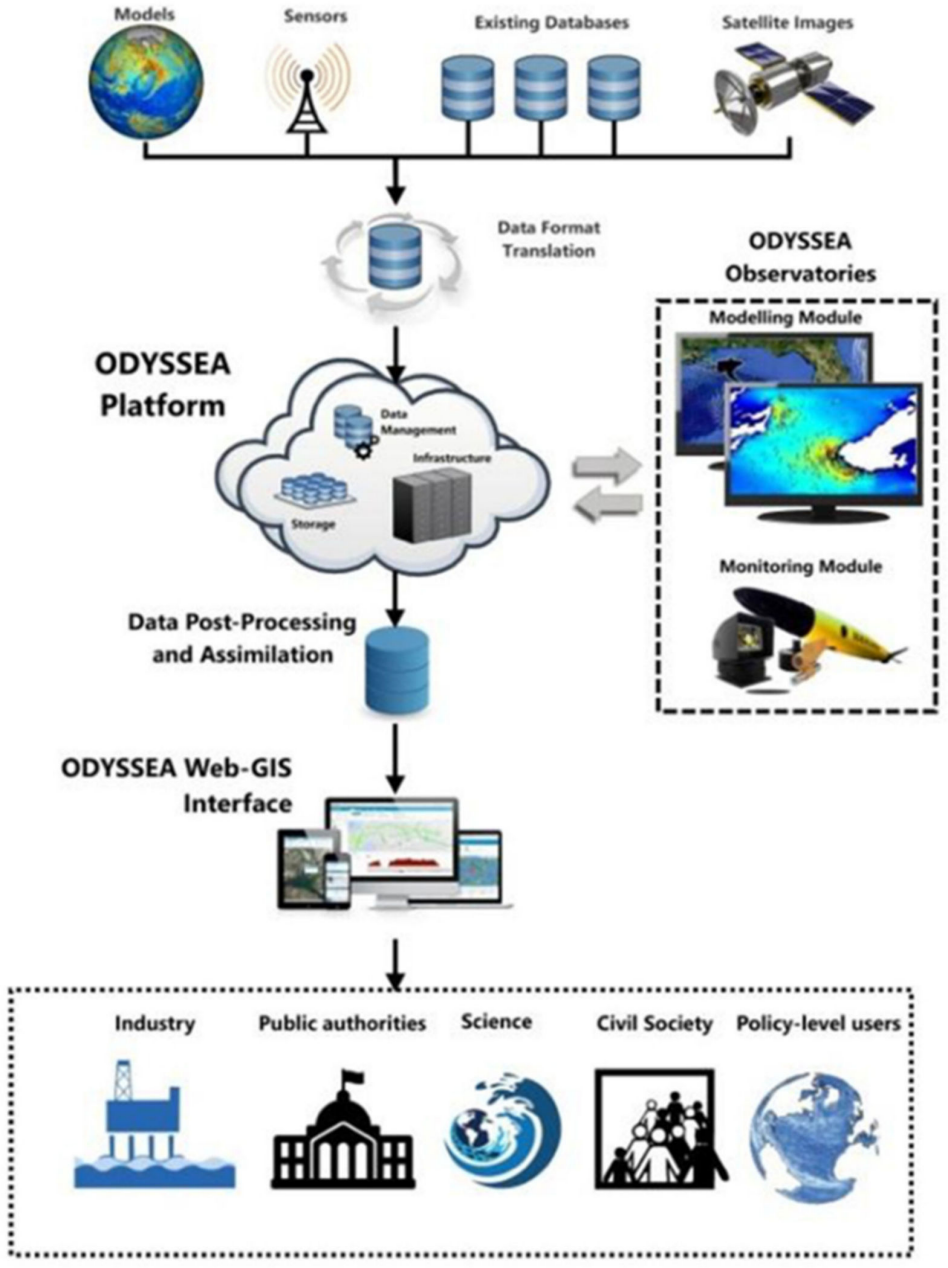
ODYSSEA Platform workflow from sensors and modeling data through the Internet and integration steps leading to post-processing and interface display for a range of decision makers and stakeholders.

Hybrid or mixed modeling approaches, that incorporate monitoring data, can also be used within integration frameworks. In this approach, real-time data are used to update forecasting models, such as watershed and water quality models. These same models complement sensor networks to fill data gaps, and provide estimates where sensor networks are not deployed or have missing data. An example of a fully integrated monitoring and modeling platform is ODYSSEA (http://odysseaplatform.eu/, accessed on July 2021) (Figure 8). The ODYSSEA platform is connected constantly with existing observational systems, models, and databases in the Mediterranean Sea, building on key initiatives, such as Copernicus, Global Earth Observation System of Systems (GEOSS), Global Ocean Observing System (GOOS), European Marine Observation and Data Network (EMODnet), among others. Further, the platform is connected to nine regional pilot observatories where novel monitoring systems are installed (to increase spatial and temporal resolution). Copernicus data and products are automatically and regularly fetched, organized, harmonized, and combined with in situ, and meteorological information from National Oceanic and Atmospheric Administration (NOAA) and distributed to enhance monitoring capabilities of water quality issues.

The integrated framework described here for global and regional scale is also implemented by other platforms at local scales. An example is the HiSea platform (http://hiseaproject.com, accessed on July 2021) that delivers high resolution information obtained through the integration of in situ, remote sensing, and modeled data and the downscaling of Copernicus and NOAA products. Copernicus provides information at a regional scale that cannot be used to properly describe the system dynamics at the local scale. This is even more true for estuaries, coastal lagoons, and inland waters. HiSea aims to remove the barriers by making information easily accessible to a broader public through access to a single platform.

Projects requiring full integration need to be chosen selectively because of limited resources in operationalizing fully integrated monitoring networks. In addition, adapting the scale of the integration will be necessary depending on the type of decision and potential impact. It takes time and experience to integrate all measures, and open exchange of lessons learned will increase the use of such an integrated framework. This integrative framework will be successful if it is transparent, practical to implement, contributes to appropriate goals that can be measured, provides flexibility, and improves decision making. Providing information and forecast capabilities of different scenarios, and their uncertainties, to decision makers enables them to identify the best actions based on solid scientific evidence.

## 5. Summary

Scaling science is a paradigm used by Gargani and McLean [153] to provide social impact for the public good. This approach underpins the GEO AquaWatch goal to develop and build the global capacity and utility of water quality data, products, and information to support water resource management and decision making. Scaling science is normally based on inclusive coordination, optimal scale, and dynamic evaluation, to transition from user ideas and needs, to knowledge and impacts. Inclusive coordination is where scientists actively engage and iterate with end-users, such as the iterative development model discussed previously. Optimal scale is acknowledging that there are trade-offs when the scale of study is too fine or too large. Dynamic evaluation is where scientists and stakeholders have a willingness to adapt to new technologies, such as CubeSats or drones, based on feedback. Moving to larger scale requires replication, where integrative components can be articulated and standardized [154]. The framework proposed in this paper includes those components.

A growing list of integrated data to information systems specific to water quality management includes NOAA’s tides and currents [155]; harmful algae bloom forecasting systems [156,157]; Spain’s Portus (https://portus.puertos.es, accessed on July 2021); EAWAG’s Swiss lake information portal (https://simstrat.eawag.ch/ for 1-D and http://meteolakes.ch/ for 3-D models, both links accessed on July 2021); the UK Lakes Portal (https://eip.ceh.ac.uk/apps/lakes, accessed on July 2021) the HAB Satellite Analysis tool from San Francisco Estuary Institute (https://fhab.sfei.org/, accessed on July 2021); Datalakes online platform for Swiss lakes (https://live.datalakesapi.ch/, accessed on July 2021); CoBIOS: Coastal Biomass Observatory Services, the high biomass blooms in Europe’s coastal waters models; and MoS2 project for southern North Sea [141]. All these systems aim at providing a synoptic view through user-friendly interfaces and information to help assess the state of water quality and, ultimately, mitigate the multiple reactions and hazards resulting from changes in conditions.

In this paper, the framework for integrating data products, derived from multiple observations and modeling, through data assimilation techniques for regional and local water quality nowcasts and forecasts, is conceptualized. It is assumed that all models, satellite algorithms, and data products are already at a satisfactory level for stakeholder needs available as interpretation ready data. Prior requirements, such as calibration, validation, and data quality control and assurance, were outside the scope of consideration here. The sustainability of various observing systems remains a major concern. However, through improved communication between scientists and stakeholders, there is an improved likelihood that scientifically sound information can be transferred and understood by stakeholders for management decisions. Stakeholder obstacles include correct and easily accessible interpretation of data into information, particularly when programming skills may be required to derive additional information, and expert knowledge is required to understand technology limitations and capabilities.

The GEO AquaWatch Initiative will continue to improve the transfer of data to information for water-quality-related decisions through integrated efforts, as conceptualized here. Moving forward, GEO AquaWatch recognizes the involvement of stakeholders at the start-up phase of any project and recommends defining integration needs based on stakeholder requirements, in addition to scientific requirements, to adequately address hypotheses. The value of information to stakeholders will be enhanced through the integration of data products in a standard approach using a common platform. This effort will enable users open access to readily available water quality data through data sharing and capacity building.

## Figures and Tables

**Table 1.** Examples of citizen science projects focused on water quality.

Citizen Science project	Website	Water quality parameter measured	Area
Bloomin’Algae	https://www.ceh.ac.uk/algal-blooms/bloomin-algae	Harmful Algal Blooms (Cyanobacteria)	UK
BloomWatch	https://cyanos.org/bloomwatch/	Harmful Algal Blooms (Cyanobacteria)	USA
CyanoTracker	http://www.cyanotracker.uga.edu/	Harmful Algal Blooms (Cyanobacteria)	USA
FreshWaterWatch	https://freshwaterwatch.thewaterhub.org/	Water Quality (Nitrate, Phosphate, Clarity)	Global
Secchi Dip In	http://www.secchidipin.org/	Water Clarity	Global
Secchi Disk	http://www.secchidisk.org/	Water Clarity	Global
The Sneaker Index	Crooke et al. [105]	Water Clarity	Chesapeake Bay (USA)
EyeOnWater	https://www.eyeonwater.org/	Water Color and Clarity	Global, Australia
HydroColor	http://misclab.umeoce.maine.edu/research/HydroColor.php); [106]	Remote-Sensing Reflectance	Global

## Data Availability

Not applicable.
